# Diagnostic accuracy of different keratoconus detection indices of pentacam in paediatric eyes

**DOI:** 10.1038/s41433-022-02070-x

**Published:** 2022-05-03

**Authors:** Ahmed Osama Hashem, Bassem Fayez Aziz, Sherine Shafik Wahba, Maged Maher Roshdy, Amr Ismail Elawamry

**Affiliations:** 1 Faculty of Medicine, Kafr El Sheikh University, Kafr El Sheikh, Egypt; 2 Faculty of Medicine, Ain Shams University, Cairo, Egypt; 3 Watany Eye Hospitals, Cairo, Egypt

**Keywords:** Corneal diseases, Eye abnormalities

## Abstract

**Objective:**

No diagnostic gold standard for keratoconus in children and adolescents exists. Our objective was investigating the diagnostic accuracy of various indices for keratoconus (KC) detection in paediatric eyes.

**Methods:**

All retrievable data of significance from 432 normal right paediatric eyes and 48 eyes of paediatric KC and forme fruste KC (FFKC), imaged by use of a rotating Scheimpflug camera (Oculyzer II, Pentacam HR) between December 2013 and October 2018 at Watany Eye Hospitals, Cairo, Egypt, including Scheimpflug images data, were collected. The area under the receiver operating characteristic curve (AUROC) was calculated for different indices in this retrospective descriptive study.

**Results:**

All 36 tested indices showed discriminative power differentiating KC and FFKC from normal corneas (AUROC *P*-value <0.05), except AC volume, AC angle, and horizontal decentrations of the steepest and thinnest points. The 32 indices showed variable degrees of diagnostic accuracy. The highest AUROC was that of the corneal assessment index from the relational thickness and other OCULUS values (CAIRO 8). Only 8 indices showed non-inferiority to it, namely, Ambrosio’s relational thickness maximum (ART max) and avg, the pachymetric progression index maximum (PPI max) and avg, the back elevation from the best-fit toric ellipsoid (BE BFTE), the KC index (KI), the topographic KC indices (TKC), and the index of height decentration (IHD) (*P* > 0.05).

**Conclusions:**

The 8 most useful rotating Scheimpflug imaging indices for KC detection in paediatric eyes are CAIRO 8 followed by ART max and avg, PPI max and avg, BE BFTE, KI, TKC, and IHD.

## Introduction

Keratoconus (KC) is an ectatic corneal disorder characterised by progressive thinning of the cornea and structural weakening, resulting in corneal protrusion, irregular astigmatism, and reduced vision [1, 2].

KC usually starts at puberty. In the paediatric age group, KC is often more advanced at the time of diagnosis and the progression of the disease is more rapid, with a higher need for corneal grafting because treatment by corneal crosslinking, the Athens protocol, intracorneal rings, or ring segments must be performed at earlier stages. Moreover, visual impairment can affect social and educational development in children [3]. Therefore, the detection of progressive KC in its early stages is necessary to prevent such consequences [4].

Few papers have discussed the epidemiology of paediatric KC, but a Lebanese study reported an incidence of 0.53% in children aged 14 years or younger who were diagnosed in a tertiary referral eye centre [5]. Based on the American Academy of Ophthalmology’s Intelligence Research in Sight Registry (IRIS), the prevalence of KC is 0.16% in the paediatric population [6].

The clinical manifestation of KC in children is somewhat different from that seen in adults. The cone is reported to be more centrally located in paediatric cases and, therefore, irregular astigmatism is less evident [7]. Clinically, the diagnosis of frank KC is straightforward. However, when it comes to subclinical, form fruste, suspect, or borderline cases, there is widespread ambiguity. The implications of tomographic indices are of the highest value and impact in these cases before the KC is frankly manifest [8–11]. The Scheimpflug camera system with its tomographic indices enables the assessment of both the anterior and posterior corneal surfaces [12]. The detection of posterior corneal surface abnormalities in clinically normal patients was a breakthrough in the diagnosis and monitoring of the disease [13].

As the minimum age approved for corneal refractive surgery is 18 years, corneal tomography is not commonly done in children and adolescents, so this age group has no well-established diagnostic gold standard [14]. We therefore published the normative data in paediatric age groups [15]. The current study aims to evaluate the accuracy and to compare the different indices of KC detection in paediatric eyes using the rotating Scheimpflug camera (Oculyzer II, Pentacam HR).

## Materials and methods

This is a retrospective observational study conducted at Watany Eye Hospitals, Cairo, Egypt. The database of cases examined in the time interval between December 2013 and October 2018 by Allegro Oculyzer II (WaveLight, GmbH, Erlangen, Germany), using the current software version 1.20r20 equivalent to Pentacam HR, was searched for paediatric cases between 3 and 18 years old [16]. The candidates with bad scan quality, previous ocular trauma, surgeries, or corneal pathology other than ectasia were excluded. All retrievable data of significance from the remaining 432 normal paediatric right eyes and 48 eyes of paediatric KC (corneas with slit-lamp signs as Vogt striae or Fleisher ring or frank tomographic diagnosis) or forme fruste KC (FFKC) (fellow eye of a frank KC but with no abnormal findings by either slit-lamp examinations or corneal tomography) were collected. The study adhered to the tenets of the Declaration of Helsinki and was approved by the Ethics Committee of Watany Eye Hospitals and waived from consent taking as it is a retrospective study including no intervention other than the usual care offered.

### The investigated indices were

A. *Anterior Chamber Parameters:*

1. Internal anterior chamber depth (ACD)
2. Anterior chamber volume at 10-mm diameter (ACV)
3. Maximum anterior chamber angle in the horizontal meridian (ACA)

B. *Curvature-based Indices:*

1. Index of height asymmetry (IHA)
2. Index of height decentration (IHD)
3. Index of surface variance (ISV)
4. Index of vertical asymmetry (IVA)
5. Central keratoconus index (CKI)
6. Keratoconus index (KI)
7. Mean curvature power of the cornea within the central 3 mm circle expressed in dioptres (K mean)
8. Keratometric power of the flat meridian (K1)
9. Keratometric power of the steep meridian (K2)
10. Keratometry of the steepest point of the front surface (K max front)
11. Steepest point of the front surface keratometry displacement in the x-axis (K max front x)
12. Steepest point of the front surface keratometry displacement in the y-axis (K max front y)
13. Keratometric astigmatism (absolute value)
14. True net power at the corneal apex (TNP apex)
15. Tilt by Fourier analysis (Tilt)
16. Mean eccentricity in the central 30 degrees by Fourier analysis (Ecc Sph)
17. High-order irregularity by Fourier analysis (other than spherical power and asphericity, tilt and astigmatism) (Irregularity)

C. *Elevation-based Indices:*

1. Front elevation from the best-fit sphere (FE from BFS)
2. Front elevation from the best-fit toric ellipsoid (FE from BFTE)
3. Back elevation from the best-fit sphere (BE from BFS)
4. Back elevation from the best-fit toric ellipsoid (BE from BFTE)

D. *Pachymetry-based Indices:*

1. Corneal thickness at the apex (Pachy apex)
2. Corneal thickness at the point corresponding to the pupil centre (Pachy pupil)
3. Corneal thickness at the thinnest point (TP)
4. Minimum pachymetric progression index (PPI min)
5. Average pachymetric progression index (PPI avg)
6. Maximum pachymetric progression index (PPI max)
7. Thinnest point displacement at the y-axes (TP y)
8. Thinnest point displacement at the x-axes (TP x)

E. *Combined Indices:*

1. Topographic keratoconus indices (TKC)
2. Ambrosio’s relational thickness average (ART avg)
3. Ambrosio’s relational thickness maximum (ART max)
4. Corneal assessment index from the relational thickness and other OCULUS values analysed for 8 mm zone (CAIRO 8) [17]

The diagnostic accuracy of an index based on the area under the receiver operating characteristics (AUROC) was stratified as either excellent (>0.9), good (0.8 to 0.9), fair (0.7 to 0.8), or poor (0.6 to 0.7). An AUROC < 0.6 is considered a “failure” and should not be used for diagnosis [18].

### Statistical analysis

Data were collected and verified, and the CAIRO 8 index was calculated. Statistical analyses were performed using MedCalc Statistical Software version 19.2.1 (MedCalc Software Ltd., Ostend, Belgium). All the retrievable data from patients fitting the inclusion and exclusion criteria gave a significantly larger sample size than that of most similar studies [19], and a post hoc analysis showed that the sample size was adequate to assess the accuracy (AUROC significantly different from the null hypothesis (0.5) for all indices of interest “PPI avg and max, ART avg and max, FE and BE from BFS [19] and most of the other indices. The following tests were performed: calculation of the mean, 95% confidence interval (CI), chi-square test, the AUROC and its binomial exact confidence interval, the AUROC comparison with the null hypothesis and with the AUROC of the most accurate index using the DeLong method [20], the best cut-off value, and the sensitivity and specificity for each index. *P*-values were considered statistically significant if less than 0.05.

## Results

The study comprised 432 normal eyes, 8 FFKC eyes, and 40 frank KC eyes. KC and FFKC were more commonly prevalent in older age groups: the mean age was 13.5 years (95% CI: 13.2 to 13.8) in normal eyes and 15.3 years (14.7 to 15.9) in KC and FFKC eyes. There was no statistically significant sex predilection (Table 1).

**Table 1:** Age and sex distribution of KC and FFKC.

		Age group			Sex	
		3–6 y	6–12 y	12–18 y	Male	Female
Normal	432 (90.0%)	17	126	289	240	192
FFKC	8 (1.7%)	0	1	7	4	4
KC	40 (8.3%)	0	4	36	22	18
		17 (3.50%)	131 (27.30%)	332 (69.20%)	266 (55.40%)	214 (44.60%)
χ^2^ test *P*-value		0.030			0.951	

**Fig. 1:**
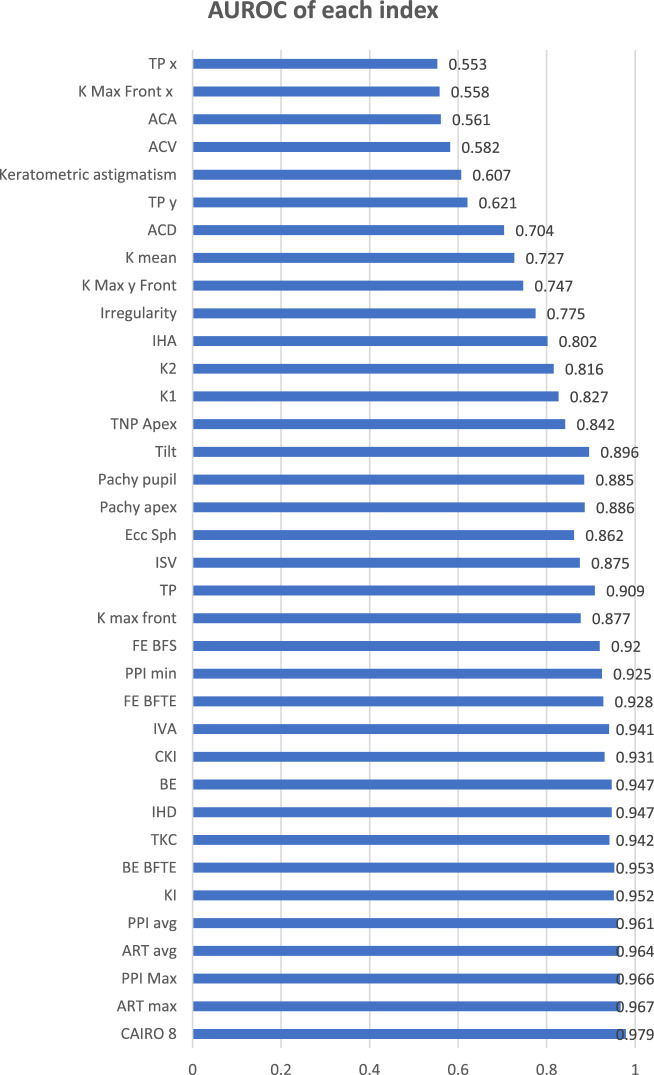
The AUROC of different tomographic indices in the studied population. TP x thinnest point displacement in the x-axis, K Max Front x steepest point of the front surface keratometry displacement in the *x*-axis, ACA maximum anterior chamber angle in the horizontal meridian, ACV anterior chamber volume at 10-mm diameter, keratometric astigmatism (absolute value), TP y thinnest point displacement in the *y*-axis, ACD internal anterior chamber depth, K mean curvature power of the cornea within the central 3-mm circle expressed in diopters, K Max y Front steepest point of the front surface keratometry displacement in the *y* axis, Irregularity high-order irregularity by Fourier analysis (other than spherical power and asphericity, tilt, and astigmatism), IHA index of height asymmetry, K2 keratometric power of the steep meridian, K1 keratometric power of the flat meridian, TNP Apex true net power at the corneal apex, Tilt tilt by Fourier analysis, Pachy pupil corneal thickness at the point corresponding to the pupil centre, Pachy apex corneal thickness at the apex, Ecc Sph mean eccentricity in the central 30 degrees by Fourier analysis, ISV index of surface variance, TP corneal thickness at the thinnest point, K max front keratometry of the steepest point of the front surface, FE BFS front elevation from the best-fit sphere, PPI min minimum pachymetric progression index, FE BFTE front elevation from the best-fit toric ellipsoid, IVA index of vertical asymmetry, CKI central keratoconus index, BE back elevation from the best-fit sphere, IHD index of height decentration, TKC topographic keratoconus indices, BE BFTE back elevation from the best-fit toric ellipsoid, KI keratoconus index, PPI avg average pachymetric progression index, ART avg Ambrosio’s relational thickness average, PPI Max maximum pachymetric progression Index, ART max Ambrosio’s relational thickness maximum, CAIRO 8 corneal assessment index from the relational thickness and other OCULUS values analysed for an 8-mm zone.

**Fig. 2:**
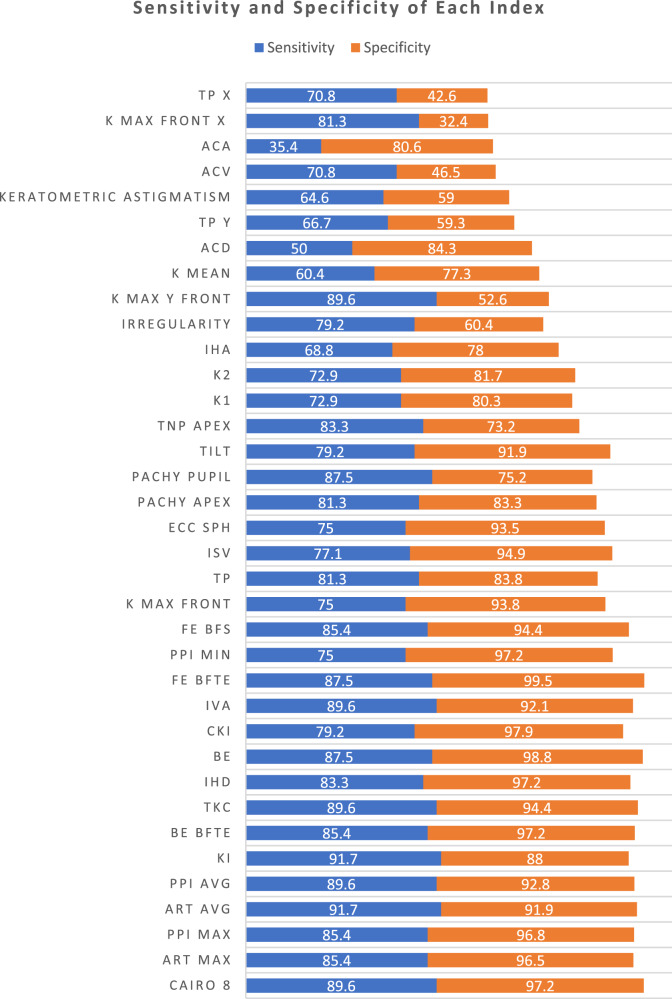
The sensitivities and specificities of all the tomographic indices. TP x thinnest point displacement in the *x*-axis, K Max Front x steepest point of the front surface keratometry displacement in the *x*-axis, ACA maximum anterior chamber angle in the horizontal meridian, ACV anterior chamber volume at 10-mm diameter, keratometric astigmatism keratometric astigmatism (absolute value), TP y thinnest point displacement in the *y*-axis, ACD internal anterior chamber depth, K mean mean curvature power of the cornea within the central 3-mm circle expressed in diopters, K Max y Front steepest point of the front surface keratometry displacement in the *y*-axis, Irregularity high-order irregularity by Fourier analysis (other than spherical power and asphericity, tilt, and astigmatism), IHA index of height asymmetry, K2 keratometric power of the steep meridian, K1 keratometric power of the flat meridian, TNP Apex true net power at the corneal apex, Tilt tilt by Fourier analysis, Pachy pupil corneal thickness at the point corresponding to the pupil centre, Pachy apex corneal thickness at the apex, Ecc Sph mean eccentricity in the central 30 degrees by Fourier analysis, ISV index of surface variance, TP corneal thickness at the thinnest point, K max front keratometry of the steepest point of the front surface, FE BFS front elevation from the best-fit sphere, PPI min minimum pachymetric progression index, FE BFTE front elevation from the best-fit toric ellipsoid, IVA index of vertical asymmetry, CKI central keratoconus index, BE back elevation from the best-fit sphere, IHD index of height decentration, TKC topographic keratoconus indices, BE BFTE back elevation from the best-fit toric ellipsoid, KI keratoconus index, PPI avg average pachymetric progression index, ART avg Ambrosio’s relational thickness average, PPI Max maximum pachymetric progression Index, ART max Ambrosio’s relational thickness maximum, CAIRO 8 corneal assessment index from the relational thickness and other OCULUS values analysed for an 8-mm zone.

**Fig. 3:**
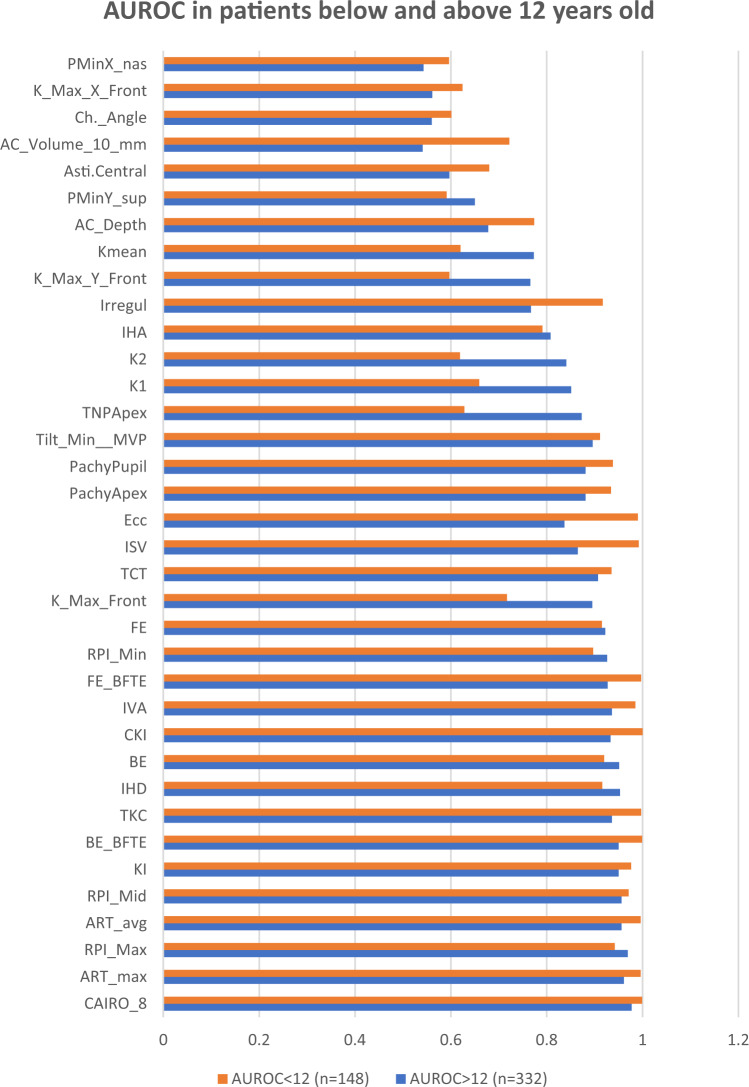
The AUROC of all the tomographic indices in patients below and above 12 years old. PMinX_nas thinnest point displacement in the *x*-axis, K_Max_X_Front steepest point of the front surface keratometry displacement in the *x*-axis, Ch._Angle maximum anterior chamber angle in the horizontal meridian, AC_Volume_10 _mm anterior chamber volume at 10-mm diameter, Asti. Central keratometric astigmatism keratometric astigmatism (absolute value), PMinY_sup thinnest point displacement in the *y*-axis, AC_Depth internal anterior chamber depth, K mean mean curvature power of the cornea within the central 3-mm circle expressed in diopters, K_Max_y_Front steepest point of the front surface keratometry displacement in the *y*-axis, Irregul high-order irregularity by Fourier analysis (other than spherical power and asphericity, tilt, and astigmatism), IHA index of height asymmetry, K2 keratometric power of the steep meridian, K1 keratometric power of the flat meridian, TNPApex true net power at the corneal apex, Tilt_Min_MVP tilt by Fourier analysis, PachyPupil corneal thickness at the point corresponding to the pupil centre, PachyApex corneal thickness at the apex, Ecc mean eccentricity in the central 30 degrees by Fourier analysis, ISV index of surface variance, TKC topographic keratoconus indices, K_max_front_keratometry of the steepest point of the front surface, FE front elevation from the best-fit sphere, RPI_Min minimum pachymetric progression index, FE_BFTE front elevation from the best-fit toric ellipsoid, IVA index of vertical asymmetry, CKI central keratoconus index, BE back elevation from the best-fit sphere, IHD index of height decentration, TKC topographic keratoconus indices, BE_BFTE back elevation from the best-fit toric ellipsoid, KI keratoconus index, RPI_Mid average pachymetric progression index, ART_avg Ambrosio’s relational thickness average, RPI_Max maximum pachymetric progression Index, ART_max Ambrosio’s relational thickness maximum, CAIRO_8 Corneal Assessment Index from the Relational thickness and other OCULUS values analysed for an 8-mm zone.

The highest AUROC was that of CAIRO 8. The remaining 35 indices had numerically lower AUROCs, with 27 of them proving to be statistically inferior to CAIRO 8. Four indices had AUROC < 0.6 and failed to discriminate KC and FFKC from normal corneas (AUROC *P*-value >0.05): AC volume, AC angle, and the horizontal decentrations of the steepest point and of the thinnest point (Table 2 and Fig. 1). The 27 indices comprised 7 indices with excellent AUROC, 10 indices with good AUROC, 4 indices with fair AUROC, 2 indices with poor AUROC, and the previously mentioned 4 indices failing to discriminate the cases (Table 2 and Fig. 1). The sensitivities and specificities of all the tomographic indices are illustrated in Table 2 Fig. 2. Also, AUROC values of all the tomographic indices in patients below and above 12 years old were compared in Fig. 3.

**Table 2:** The values of the 36 indices in ectatic and non-ectatic eyes and their diagnostic accuracy.

		Normal eyes (432)		KC and FFKC (48)		Diagnostic Accuracy						
		Mean	95% CI	Mean	95% CI	AUC	95% CI	Youden criterion	95% CI	Sensitivity	Specificity	*P* from CAIRO 8
1	**CAIRO 8**	−2.51	−2.77 to −2.25	12.87	10.44 to 15.30	0.979	0.962 to 0.990	>2.71	>1.36 to >6.67	89.6	97.2	
2	**ART max**	461.6	452.1 to 471.1	204.8	179.7 to 229.9	0.967	0.947 to 0.981	≤296	≤259 to ≤366	85.4	96.5	0.346
3	**PPI max**	1.206	1.181 to 1.230	2.583	2.296 to 2.871	0.966	0.946 to 0.981	>1.7	>1.3 to >1.8	85.4	96.8	0.327
4	**ART avg**	578.6	568.3 to 588.8	283	250.6 to 315.4	0.964	0.943 to 0.979	≤443	≤428 to ≤443	91.7	91.9	0.213
5	**PPI avg**	0.95	0.935 to 0.965	1.829	1.647 to 2.012	0.961	0.939 to 0.976	>1.1	>1 to >1.2	89.6	92.8	0.188
6	**KI**	1.021	1.019 to 1.023	1.17	1.139 to 1.201	0.952	0.929 to 0.969	>1.04	>1.03 to >1.05	91.7	88.0	0.162
7	**BE from BFTE**	1.5	1.3 to 1.8	35.4	28.2 to 42.5	0.953	0.930 to 0.970	>3	>2 to >6	85.4	97.2	0.129
8	**TKC**	0.0	0.0 to 0.1	1.9	1.6 to 2.1	0.942	0.917 to 0.961	>0	>0 to >0.5	89.6	94.4	0.079
9	**IHD**	0.0115	0.0109 to 0.0121	0.069	0.0547 to 0.0833	0.947	0.923 to 0.965	>0.025	>0.019 to >0.027	83.3	97.2	0.071
10	**BE**	7.0	6.5 to 7.4	40.2	34.3 to 46.2	0.947	0.923 to 0.965	>18	>15 to >25	87.5	98.8	0.047
11	**CKI**	1.01	1.009 to 1.010	1.061	1.048 to 1.075	0.931	0.905 to 0.952	>1.02	>1.01 to >1.02	79.2	97.9	0.023
12	**IVA**	0.161	0.153 to 0.168	0.641	0.536 to 0.745	0.941	0.915 to 0.960	>0.26	>0.23 to >0.32	89.6	92.1	0.020
13	**FE from BFTE**	0.8	0.7 to 0.9	14.2	11.1 to 17.3	0.928	0.901 to 0.949	>11	>6 to >13	87.5	99.5	0.018
14	**PPI min**	0.659	0.646 to 0.673	1.398	1.221 to 1.575	0.925	0.898 to 0.947	>0.9	>0.7 to >1	75.0	97.2	0.007
15	**FE from BFS**	4.4	4.2 to 4.7	19.4	16.1 to 22.7	0.92	0.893 to 0.943	>8	>7 to >10	85.4	94.4	0.007
16	**K max front**	45.763	45.573 to 45.953	54.184	51.973 to 56.395	0.877	0.844 to 0.905	>48.82	>46.9 to >49.34	75.0	93.8	0.002
17	**TP**	534.9	531.2 to 538.5	450.271	435.0 to 465.5	0.909	0.880 to 0.934	≤500	≤476 to ≤521	81.3	83.8	0.001
18	**ISV**	30.1	29.2 to 31.0	72.4	62.1 to 82.7	0.875	0.842 to 0.903	>45	>39 to >46	77.1	94.9	0.001
19	**Ecc Sph**	0.671	0.660 to 0.683	0.962	0.892 to 1.032	0.862	0.828 to 0.892	>0.81	>0.79 to >0.88	75.0	93.5	0.001
20	**Pachy apex**	536.9	532.0 to 541.7	460.1	444.5 to 475.8	0.886	0.855 to 0.913	≤505	≤490 to ≤520	81.3	83.3	0.001
21	**Pachy pupil**	538.1	534.5 to 541.7	463.6	448.4 to 478.7	0.885	0.853 to 0.912	≤514	≤497 to ≤519	87.5	75.2	0.001
22	**Tilt**	0.234	0.222 to 0.245	0.658	0.560 to 0.756	0.896	0.865 to 0.921	>0.41	>0.39 to >0.5	79.2	91.9	<0.001
23	**TNP apex**	42.49	42.24 to 42.74	47.3	45.87 to 48.74	0.842	0.807 to 0.874	>43.6	>42.9 to >44	83.3	73.2	<0.001
24	**K1**	42.348	42.182 to 42.514	46.342	45.086 to 47.597	0.827	0.790 to 0.860	>43.7	>42.6 to >45.5	72.9	80.3	<0.001
25	**K2**	45.157	44.978 to 45.337	50.121	48.475 to 51.767	0.816	0.779 to 0.850	>46.8	>45.6 to >48.5	72.9	81.7	<0.001
26	**IHA**	6.55	6.02 to 7.08	25.10	18.11 to 32.10	0.802	0.763 to 0.837	>9.9	>4.1 to >18	68.8	78.0	<0.001
27	**Irregularity**	0.0206	0.0197 to 0.0215	0.0386	0.0311 to 0.0460	0.775	0.735 to 0.812	>0.02	>0.018 to >0.03	79.2	60.4	<0.001
28	**K Max front y**	0.391	0.269 to 0.513	−0.721	−0.969 to −0.474	0.747	0.706 to 0.786	≤0.19	≤−0.13 to ≤1.17	89.6	52.6	<0.001
29	**K mean**	43.906	43.722 to 44.090	47.141	45.707 to 48.574	0.727	0.685 to 0.767	>45.15	>43.8 to >46.05	60.4	77.3	<0.001
30	**ACD**	3.106	3.077 to 3.136	3.345	3.219 to 3.471	0.704	0.661 to 0.745	>3.41	>3.05 to >3.65	50.0	84.3	<0.001
31	**TP y**	−0.372	−0.398 to −0.346	−0.501	−0.600 to −0.403	0.621	0.576 to 0.665	≤−0.44	≤−0.53 to ≤ −0.07	66.7	59.3	<0.001
32	**Keratometric astigmatism**	2.81	2.68 to 2.94	3.78	3.03 to 4.53	0.607	0.562 to 0.651	>3.1	>0.7 to >4.4	64.6	59.0	<0.001
33	**ACV**	185.9	182.50 to 189.30	194.57	183.84 to 205.30	0.582	0.536 to 0.627	>179	>115 to >235.3	70.8	46.5	<0.001
34	**ACA**	37.89	37.24 to 38.53	39.23	36.92 to 41.54	0.561	0.516 to 0.606	>42.8	>32.2 to >46.2	35.4	80.6	<0.001
35	**K Max front x**	−0.108	−0.142 to −0.0744	−0.0281	−0.121 to 0.0643	0.558	0.513 to 0.603	>−0.2	>−0.61 to >0.07	81.3	32.4	<0.001
36	**TP x**	−0.566	−0.599 to −0.533	−0.526	−0.597 to −0.455	0.553	0.508 to 0.598	>−0.65	>−0.91 to > −0.42	70.8	42.6	<0.001

*CAIRO 8* Corneal Assessment Index from the Relational thickness and other OCULUS values analysed for an 8-mm zone, *ART max* Ambrosio’s relational thickness maximum, *PPI Max* maximum pachymetric progression Index, *ART avg* Ambrosio’s relational thickness average, *PPI avg* average pachymetric progression index, *KI* keratoconus index, *BE BFTE* back elevation from the best-fit toric ellipsoid, *TKC* topographic keratoconus indices, *IHD* index of height decentration, *BE* back elevation from the best-fit sphere, *CKI* central keratoconus index, *IVA* index of vertical asymmetry, *FE BFTE* front elevation from the best-fit toric ellipsoid, *PPI min* minimum pachymetric progression index, *FE BFS* front elevation from the best-fit sphere, *K max front* keratometry of the steepest point of the front surface, *TP* corneal thickness at the thinnest point, *ISV* index of surface variance, *Ecc Sph* mean eccentricity in the central 30 degrees by Fourier analysis, *Pachy apex* corneal thickness at the apex, *Pachy pupil* corneal thickness at the point corresponding to the pupil centre, *Tilt* tilt by Fourier analysis, *TNP Apex* true net power at the corneal apex, *K1* keratometric power of the flat meridian, *K2* keratometric power of the steep meridian, *IHA* index of height asymmetry, *Irregularity* high-order irregularity by Fourier analysis (other than spherical power and asphericity, tilt, and astigmatism), *K Max y Front* steepest point of the front surface keratometry displacement in the y-axis, *K mean* mean curvature power of the cornea within the central 3-mm circle expressed in diopters, *ACD* internal anterior chamber depth, *TP y* thinnest point displacement in the y-axis, *keratometric astigmatism* keratometric astigmatism (absolute value), *ACV* anterior chamber volume at 10-mm diameter, *ACA* maximum anterior chamber angle in the horizontal meridian, *K Max Front x* steepest point of the front surface keratometry displacement in the x-axis, *TP x* thinnest point displacement in the x-axis.

## Discussion

Paediatric KC is considered a dilemma in comparison with the adult disease, due to under-diagnosis, poor compliance with contact lens use, and modifications in treatment patterns [21].

Rotating Scheimpflug imaging is a useful tool for unveiling the disease in adults. However, there are not enough studies of paediatric KC [22] and there is a need to investigate the accuracy and the cut-off values of each index in KC diagnosis. Our study therefore evaluated these issues, revealing that the most useful rotating Scheimpflug imaging indices for KC detection in paediatric eyes were CAIRO 8, ART max and avg, PPI max and avg, BE BFTE, KI, TKC, and IHD respectively. On the other hand, four indices (ACV, ACA, K Max Front x, and TP x) did not show sufficient discriminative power between FFKC, KC, and normal corneas and can be excluded from the screening analysis.

The CAIRO 8 index is calculated by regression analysis that combines the highly sensitive ART max with the highly specific anterior elevation [17]. It was initially used for adult corneas and has now been validated in this study for paediatric corneas.

In the current study, where we analysed only data obtained from good quality scans with an analysable area of at least 9 mm^2^, analysis of elevation and pachymetry indices showed that CAIRO 8 had the highest accuracy (AUROC = 0.979) followed by ART max (AUROC = 0.967).

Our findings also support the importance of another combined index, namely ART max, which is calculated as the ratio between the thinnest point and the PPI max [23].

The results show that the pachymetric indices, namely PPI max and PPI avg, come next to the combined indices in terms of higher sensitivity in paediatric eyes (AUROC = 0.966 and 0.961, respectively). Further analysis of pachymetric indices revealed that thickness at TP had a higher AUROC than the pachy apex and pachy pupil. This can be attributed to the relative sensitivity to fixation of both pachy apex and pachy pupil due to the potential error of their localisation, especially in children [24]. On the other hand, the determination of the TP is not related to a certain location but rather done by scanning all available points and choosing the thinnest of them irrespective of its location. Likewise, the TP value had a higher AUROC than the AUROC of its vertical and horizontal decentrations (AUROC = 0.909, 0.621, and 0.553, respectively).

Regarding the elevation parameters, we concluded that the most accurate indices were BE BFTE (AUROC = 0.953) followed by BE, FE BFTE, and FE (AUROC = 0.947, 0.928, and 0.92, respectively). The elevations of the posterior surface, being the site of primary subclinical tomographic changes, have slightly better AUROCs than those of the anterior surface that could be partially neutralised by the epithelium [25–28].

In the paediatric population, curvature indices regain their diagnostic importance and compete with elevation indices [19, 29]. The KI, IHD, and TKC indices were among those with excellent AUROC (0.952, 0.947, and 0.942, respectively).

Although KC in the paediatric age was reported to be mainly central, the KI had higher AUROC than CKI, following the general rule in adult KC shown by Orucoglu et al. [30]. As shown by the high AUROC of IHD and KI in our study, vertical asymmetry seems prevalent in paediatric cases.

K max front was more accurate than its vertical and horizontal decentrations (AUROC = 0.877 versus 0.747, 0.558) and the K mean, (AUROC = 0.877 versus 0.727).

Aslankurt et al. compared 52 eyes of children with Down syndrome to 60 eyes of normal children regarding early topographic changes. They found that ISV had the highest sensitivity for subclinical KC (AUROC = 0.887) [24]. In the current study, although the ISV AUROC was 0.875, which is good and comparable to the results of Aslankurt, it was still significantly inferior to CAIRO 8 (*P* = 0.001). The ISV reflects the variation of the corneal radii of curvature compared with the mean value. Therefore, it is simply a non-specific expression of the surface curvature irregularity, including non-ectatic high-corneal astigmatism [18, 31].

In children, the corneal ectasia may have enough amplitude to affect the central ACD (AUROC = 0.704), but not yet enough widespread corneal involvement to affect the AC volume and the AC angle (0.582 and 0.561, respectively).

To the best of our knowledge, this is the first study to investigate the accuracy of different Pentacam indices in the detection of KC in a wide range of ages of paediatric eyes and with a relatively large sample size. The limitation of this study is its retrospective nature. Therefore, we recommend future prospective longitudinal studies for those indices. On the other hand, we also recommend further study of paediatric corneas biomechanics.

In conclusion, for diagnosis of paediatric KC or even FFKC, we recommend relying on some indices, namely CAIRO 8, ART max and avg, PPI max and avg, BFTE, KI, TKC, and IHD, rather than the other indices.

### Summary

#### What was known before:

- Pentacam is a reliable tool for early detection of KC in adults.
- Paediatric age group has no well-established diagnostic gold-standard of corneal ectasia.

#### What this study adds:

- The current study evaluated the accuracy of different tomographic indices of KC in paediatric eyes and found that Pentacam can accurately diagnose KC in paediatric age group
- We recommended the use of the CAIRO 8, ART max and avg, PPI max and avg, BFTE, KI, TKC, and IHD indices.

## Supplementary information

Supplemental Material - Patients's data excel sheet

## Data Availability

The datasets generated during and analysed during the current study are available from the corresponding author on reasonable request.
